# In Vitro Characterization of Human Cell Sources in Collagen Type I Gel Scaffold for Meniscus Tissue Engineering

**DOI:** 10.3390/gels10120767

**Published:** 2024-11-25

**Authors:** Barbara Canciani, Nicolò Rossi, Elena Arrigoni, Riccardo Giorgino, Mirko Sergio, Lucia Aidos, Mauro Di Giancamillo, Valentina Rafaela Herrera Millar, Giuseppe M. Peretti, Alessia Di Giancamillo, Laura Mangiavini

**Affiliations:** 1 IRCCS Ospedale Galeazzi-Sant’Ambrogio, 20157 Milan, Italy; canciani.barbara@hsr.it (B.C.); nicolo.rossi@grupposandonato.it (N.R.); giuseppe.peretti@unimi.it (G.M.P.); laura.mangiavini@unimi.it (L.M.); 2 Department of Biomedical Sciences for Health, University of Milan, 20141 Milan, Italy; elena.arrigoni@hotmail.it (E.A.); valentina.herrera@unimi.it (V.R.H.M.); 3 Residency Program in Orthopedics and Traumatology, University of Milan, 20141 Milan, Italy; riccardo.giorgino@unimi.it; 4 Department of Veterinary Medicine and Animal Science, University of Milan, 26900 Lodi, Italy; mirko.sergio@unimi.it (M.S.); lucia.aidos@unimi.it (L.A.); mauro.digiancamillo@unimi.it (M.D.G.)

**Keywords:** mesenchymal stem cells, dermal fibroblasts, growth factors, collagen scaffold, gel scaffold, meniscal replacement, meniscal tissue engineering

## Abstract

Strategies to repair the meniscus have achieved limited success; thus, a cell-based therapy combined with an appropriate biocompatible scaffold could be an interesting alternative to overcome this issue. The aim of this project is to analyze different cell populations and a collagen gel scaffold as a potential source for meniscus tissue engineering applications. Dermal fibroblasts (DFs) and mesenchymal stem cells (MSCs) isolated from adipose tissue (ASCs) or bone marrow (BMSCs) were analyzed. Two different fibro-chondrogenic media, M1 and M2, were tested, and qualitative and quantitative analyses were performed. Significant increases in glycosaminoglycans (GAGs) production and in fibro-cartilaginous marker expression were observed in MSCs in the presence of M1 medium. In addition, both ASCs and BMSCs cultured in M1 medium were used in association with the collagen hydrogel (MSCs-SCF) for the development of an in vitro meniscal-like tissue. Significant up-regulation in GAGs production and in the expression of aggrecan, collagen type I, and collagen type II was observed in BMSCs-SCF. This study improves knowledge of the potential of combining undifferentiated MSCs with a collagen gel as a new tissue engineering strategy for meniscus repair.

## 1. Introduction

In the orthopedic field, the meniscus plays a crucial role in knee function and biomechanics, particularly due to its anatomy and biological components. Fibrochondrocytes represent the cellular component of the meniscus and can secrete the fibrocartilaginous matrix [1]. This matrix is mainly composed of type I collagen and, in a minor amount of collagens (types II–VI), aggrecans, and glycosaminoglycans (GAGs), which contribute to meniscus resistance, functional structure, and biomechanics [2]. Given the important role of this tissue, lesions or diseases that alter its structure can significantly impact the quality of life. Lesions of the meniscus are frequently observed in orthopedic practice occurring both in younger and elderly patients either caused by minimal twisting or stress or by chronic degenerative processes [3]. Many treatments for an injured meniscus have been described, including enhancement of surgical repair by fibrin clots or meniscus grafting after meniscectomy using allografts or tendons [4,5]. Therefore, there has been increasing scientific and clinical interest in meniscus tissue engineering aimed at minimizing the risk of developing knee osteoarthritis and offering a solution for patients suffering from persistent symptoms post-meniscectomy [6]. In recent decades, tissue engineering approaches have taken center stage in biomedical research to improve the reparative processes of joint tissues throughout the development of a tissue-engineered construct. The possibility of entirely reproducing the meniscus structure and function is highly attractive and relies on the fine-tuning of biological and biophysical cues of selected cells seeded on a biomimetic scaffold [7,8]. Since pioneers first started, numerous biomaterials have been used to produce such a substitute, from autologous tissue to synthetic materials [9,10,11,12]. The use of scaffolds in meniscal tissue engineering is pivotal for achieving successful regeneration of meniscal tissues, particularly due to their interaction with various cell sources [4]. Understanding this interaction enhances the design of scaffolds and optimizes their application in regenerative medicine, as already reviewed by Suamte et al. [13]. Avitene™ UltraFoam™ sponge is a gelatin-based hemostatic agent with a microfibrillar collagen structure, proven to accelerate clot formation due to its active absorbable collagen hemostat [14]. The ultrastructure appears porous, indicating that the sponge might be an ideal scaffold for cartilage/meniscus tissue engineering. Another important element in tissue engineering approaches is identifying the most suitable cell source. Several cell populations have been tested for meniscus repair and regeneration, such as articular chondrocytes [15] and fibrochondrocytes [16,17], and histological evidence highlighted the ability of these cell populations to generate fibrocartilaginous tissue resembling the meniscus. However, due to the difficulty of harvesting a sufficient number of cells, autologous fibrochondrocytes or chondrocytes do not represent optimal cell sources in tissue engineering approaches. These problems have driven scientists to search for alternative cell populations capable of acquiring a fibrochondrocyte-like phenotype after treatment with specific growth factors [18,19]. Mesenchymal stem cells (MSCs) are considered promising candidates in tissue engineering applications, due to their high therapeutic potential: they are easily isolated from several adult tissues, such as bone marrow and adipose tissue; they possess a good proliferative potential; and they can differentiate into several cell lineages including osteoblasts, chondrocytes, and adipocytes [20,21,22,23].

Moreover, the recruitment of MSCs in tissue repair, their ability to home toward an in vivo injured site, and their low immunogenicity position them as key players in the transplantation field [24]. In addition, dermal fibroblasts (DFs) represent another eligible cell source for meniscus engineering. Previous research has demonstrated that dermal fibroblasts possess chondrogenic differentiation potential when properly stimulated [25] or in 3D co-culture with chondrocytes [26]. In this study, we compared the fibro-chondrogenic differentiation potential of MSCs isolated from both bone marrow (BMSCs) and adipose tissue (ASCs), as well as dermal fibroblasts (DFs). Specifically, we tested these three cell populations under two different culture conditions, selected from the literature with minor modifications, concerning their proliferation and differentiation capacity using both micro-mass pellet and 3D collagen scaffold models.

## 2. Results and Discussion

### 2.1. Cell Isolation and Characterization

**Figure 1:**
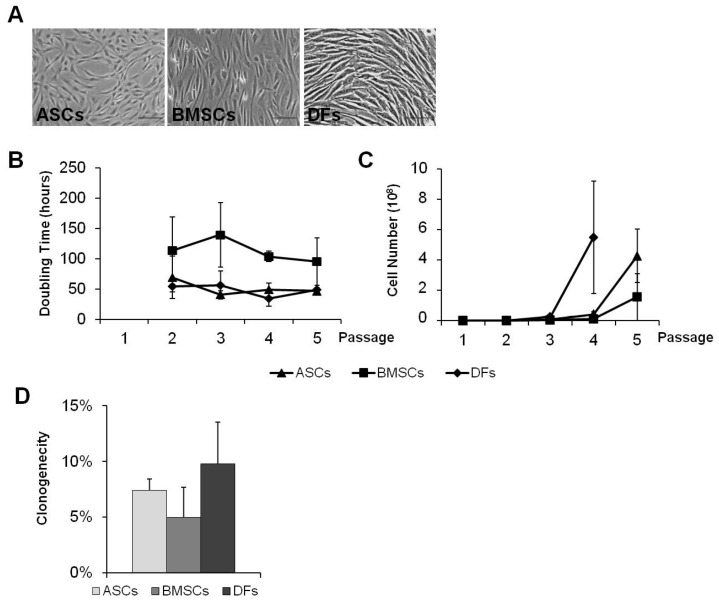
(**A**): Microscopic images showing the fusiform morphology of the three cell populations (MSCs, ASCs, and DFs) cultured under standard conditions. (**B**): Graph illustrating the doubling time (DT) of the three cell populations in culture, showing that ASCs and DFs have a lower DT compared to BMSCs. (**C**): Graph representing the total number of cells obtained after one month of culture for each cell population. ASCs and DFs show higher proliferation compared to BMSCs. (**D**): Graph showing the clonogenic capacity (CFU-F) of the three cell populations. Each cell population maintained good clonogenic capacity. Scalebar: 20 µm.

In culture, in standard conditions, the three cell populations showed a fusiform shape, also known as fibroblast-like morphology (Figure 1A). After one week of culture, MSCs, and DFs, rapidly started to proliferate (Figure 1B,C): in detail, both ASCs and DFs showed a similar trend, with an average doubling time (DT) of about 51.2 ± 6.1 h and 48.4 ± 4.9 h, respectively (Figure 1B), allowing the culture to reach, 4.3 × 10^8^ ± 1.8 × 10^8^ ASCs and 5.5 × 10^8^ ± 3.7 × 10^8^ DFs after one month, starting from 5 × 10^4^cells (Figure 1C). In contrast, BMSCs showed a lower DT compared to ASCs (104.0 ± 5.3 h) (Figure 1B) and, after 30 days of culture, they presented a cellular yield of 1.6 × 10^8^ ± 1.5 × 10^8^ (Figure 1C). The cells also demonstrated good clonogenic ability, which was maintained from the 1st to 4thpassage: specifically, about 7.9 ± 0.9, 6.0 ± 1.7 and 9.8 ± 3.8, for ASCs, BMSCs and DFs, respectively, produced CFU-F (Figure 1D).

### 2.2. Cells Differentiation Assessment

**Figure 2:**
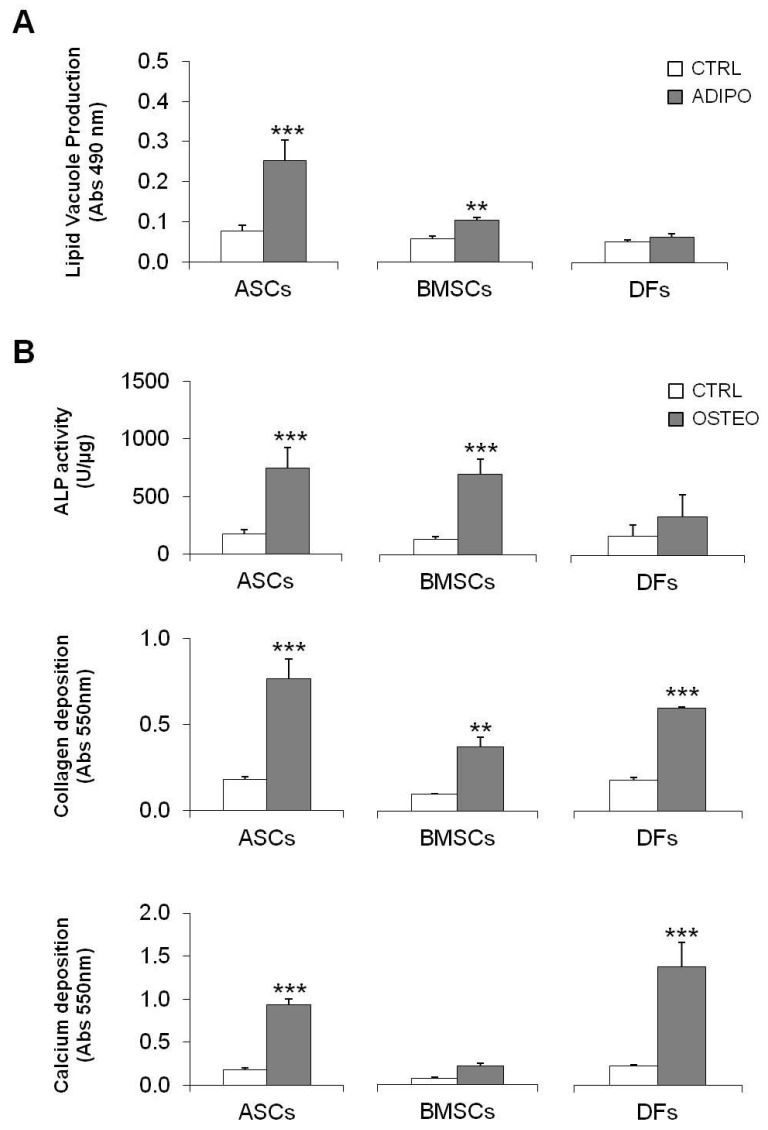
(**A**): Graph showing the induction of lipid vacuoles in MSCs after 14 days of insulin treatment. ASCs and BMSCs showed a significant increase in lipid production, while DFs showed no induction. (**B**) (**Upper Panel**): Graph representing alkaline phosphatase (ALP) activity in MSCs and DFs induced toward the osteogenic lineage. A significant increase in ALP activity was observed in ASCs and BMSCs compared to DFs. (**B**) (**Middle Panel**): Graph showing collagen production in osteo-induced cells, with significant increases in all cell populations. (**B**) (**Lower Panel**): Graph representing calcium deposition in osteo-induced cells, showing a significant increase for ASCs, BMSCs, and DFs compared to controls. **: *p* < 0.01; ***: *p* < 0.001.

Adipo-induced MSCs produced lipid vacuoles when treated for 14 days in the presence of insulin: significant increases of 230.8 (*p* < 0.001) and 81.1% (*p* < 0.01) were observed for ASCs and BMSCs, respectively (Figure 2A), whereas no induction was observed for DFs (*p* > 0.05).

In the presence of specific osteo-stimuli, both MSCs and DFs were able to differentiate towards osteogenic lineage: a significant increase of about 324.3% (*p* < 0.001) and 420.8% (*p* < 0.001) in alkaline phosphatase (ALP) activity was present in osteo-ASCs and BMSCs, while a lesser increase was observed in DFs (+94.0%, *p* > 0.05) (Figure 2B, upper panel). Further confirmation of their differentiation capacity was obtained by collagen quantification (+327.3% for ASCs, +282.1% for BMSCs, and +229.0% for DFs) (Figure 2B, middle panel) and calcium content quantification (+420.7, +197.3 e +500.8% for ASCs, BMSCs and DFs, respectively) (Figure 2B, lower panel), compared to CTRL cells.

**Figure 3:**
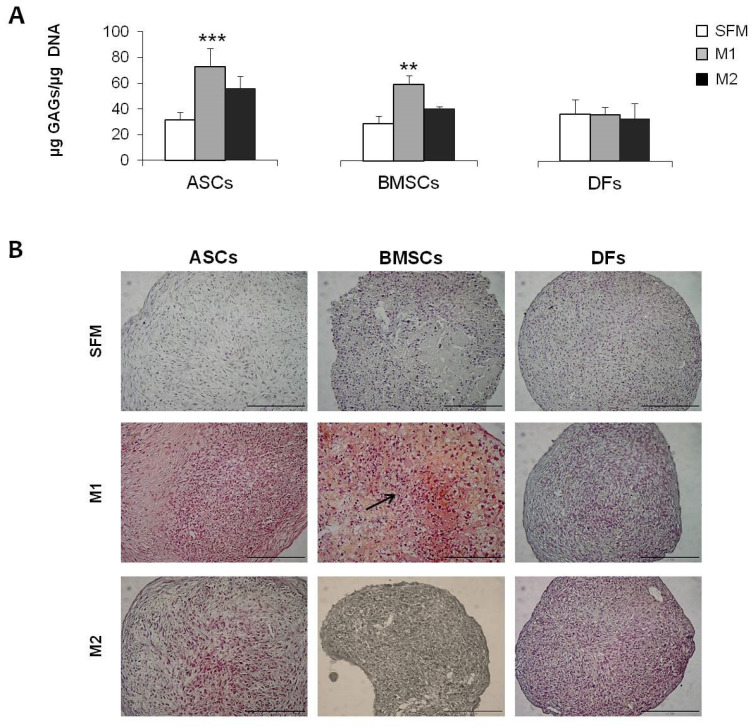
(**A**): Graph showing GAG production in MSCs and DFs cultured in pellet and treated with M1 and M2 media. A significant increase in GAGs is observed in the presence of M1 medium for ASCs and BMSCs. (**B**): Histological image showing Safranin O staining in ASCs, BMSCs, and DFs pellets treated with SFM, M1, and M2 medium. Scalebar: 200 µm. **: *p* < 0.01; ***: *p* < 0.001.

**Figure 4:**
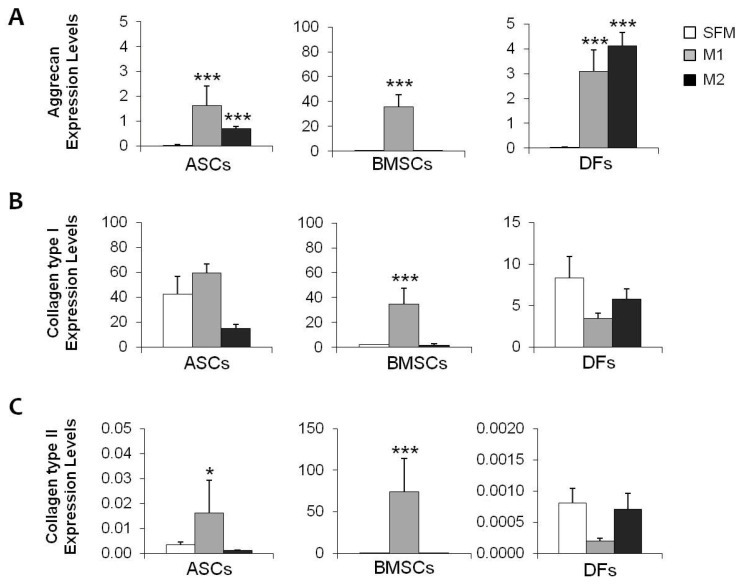
(**A**–**C**): Graphs showing gene expression of Aggrecan, Collagen type I, and Collagen type II in cell populations treated with SFM, M1, and M2 medium. *: *p* < 0.01; ***: *p* < 0.001.

MSCs and DFs were also induced for 21 days to differentiate towards fibro-chondrogenic lineage in pellet culture conditions using two inductive media. All the cell populations were able to aggregate forming micromasses. As reported in Table 1, MSCs, and, in particular, BMSCs, grew in the presence of chondro M1 medium, and showed larger dimensions, suggesting a higher extracellular matrix deposition. These results were confirmed by both biochemical and histological analysis. Significant increases in GAGs production were observed in MSCs in the presence of M1 medium (+130.9% for ASCs and +105.5% for BMSCs), and less using M2 medium (+77.1% and +41.2%, respectively) (Figure 3A). These data were also confirmed by histological evaluation: in fibro-chondro M1 BMSCs, an extracellular matrix depot was highlighted by the positive staining for Safranin O, indicating the presence of GAGs (Figure 3B) and the acquisition of chondrogenic phenotype for the differentiated cells. In addition, gene expression analysis showed a significant up-regulation of three important markers of meniscal tissue: Aggrecan, Collagen type I, and Collagen type II, in both fibro-chondro M1 ASCs and BMSCs (Figure 4A–C), compared to the same cells maintained in undifferentiated conditions. No induction towards a meniscal phenotype was observed in DFs (Figure 4).

Starting from these results, the next experiments were performed using MSCs (ASCs and BMSCs) stimulated with fibro-chondro medium M1.

### 2.3. Cells-Scaffold Interaction

The viability of MSCs isolated from both adipose tissue and bone marrow and maintained until 14 days in the presence of the scaffold, was not influenced (data not shown): a similar proliferation rate was observed for both cells cultured in monolayer (MONO) or grown in the presence of collagen (SCAFFOLD), suggesting the biocompatibility of the scaffold used. 

**Figure 5:**
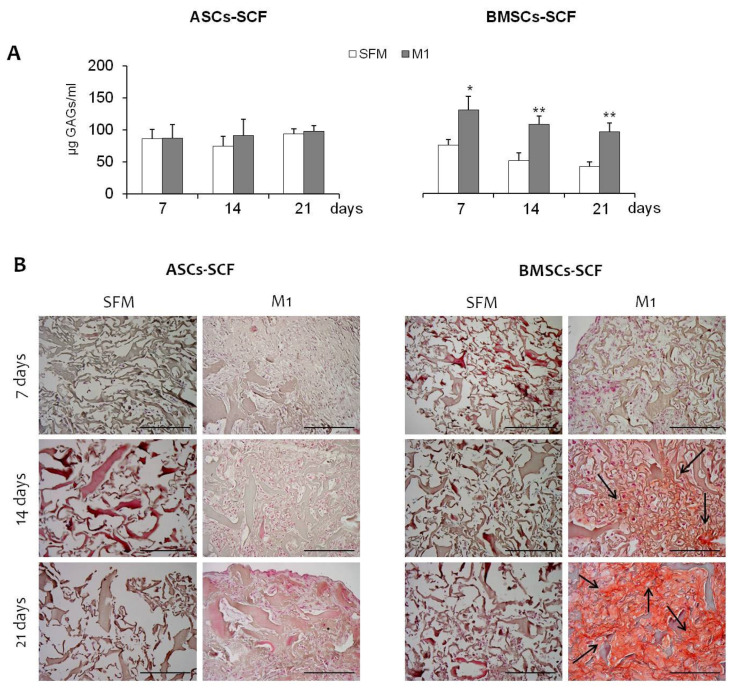
(**A**) (**Left Panel**): Graph illustrating GAG production in differentiated ASCs-SCF, with no significant increases compared to controls (SFM). (**A**) (**Right Panel**): Graph showing the increase in GAG production in differentiated BMSCs-SCF in the presence of M1 medium for 7, 14, and 21 days, compared to controls (SFM). (**B**): Histological images showing Safranin O staining in constructs generated with ASCs and BMSCs. The image highlights extracellular matrix accumulation and a more compact structure in the differentiated constructs, compared to SFM controls. Scalebar: 200 µm. *: *p* < 0.05; **: *p* < 0.01.

**Figure 6:**
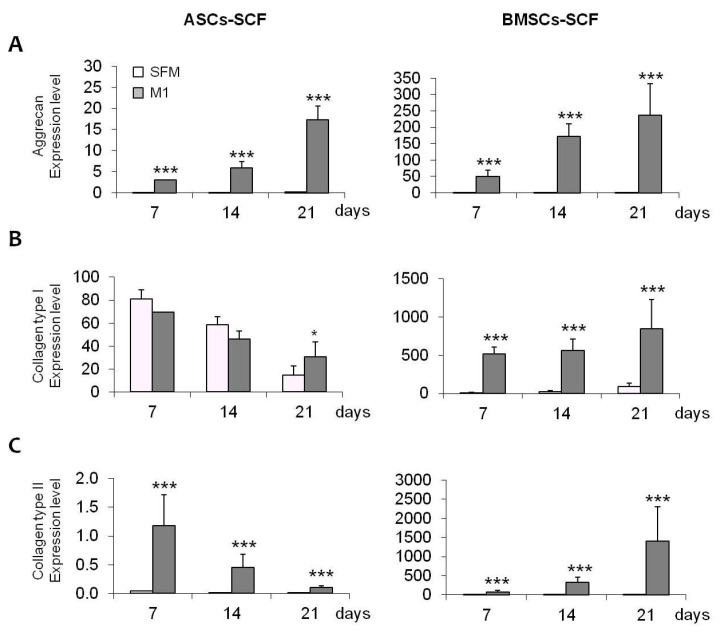
(**A**): Graph representing the upregulation of Aggrecan expression in differentiated MSCs (ASCs and BMSCs) in the presence of M1 medium, with an increase over time. (**B**,**C**) (**Left Panel**): Graphs showing Collagen type I and II expression in ASCs, with no induction for Collagen type I and a different trend for Collagen type II. (**B**,**C**) (**Right Panel**): Graphs showing Collagen type I and II expression in BMSCs in the presence of M1 medium, with a significant increase compared to controls. *: *p* < 0.05; ***: *p* < 0.001.

**Figure 7:**
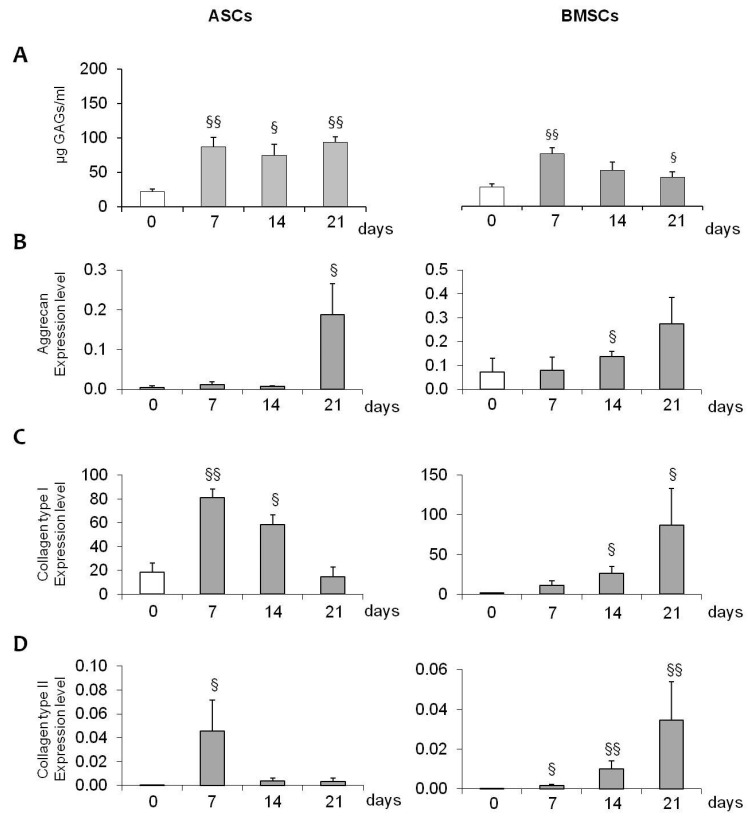
Graphs showing the effect of the collagen sponge on undifferentiated cell populations (SFM ASCs- and BMSCs-SCF). All markers evaluated for fibro-chondrogenic differentiation show a significant increase in SFM bioconstructs maintained for 7, 14, and 21 days compared to day 0, suggesting the inductive potential of the collagen sponge on MSCs. §: *p* < 0.05; §§: *p* < 0.01.

A differentiative kinetic was performed: the constructs MSCs + SCF were maintained for 7, 14, and 21 days in the absence (SFM) or in the presence of fibro-chondrogenic inductive stimuli (M1 GAG production in differentiated BMSCs-SCF increased significantly by about 95.5%, 175.3%, and 248.6% after 7, 14, and 21 days, respectively, compared to SFM-biocontrols) (Figure 5A, right panel). No statistically significant increases were observed in differentiated ASCs-SCF (+1.2, +31.7, and +6.1%, respectively, during 7, 14, and 21 days of culture) (Figure 5A, left panel). Moreover, these data were also supported by histological evaluation using Safranin-O: in Figure 5B, a significant extracellular matrix accumulation was detected for the construct generated using BMSCs. Interestingly, in both ASCs and BMSCs, during fibro-chondrogenic differentiation, the constructs became more compact and well-organized compared to those maintained in SFM, where ECM was not present, and the cells were lost (Figure 5B). In fibro-chondro MSCs-SCF, and, in particular, when BMSCs were tested, the staining for GAGs, and so the accumulation of ECM, increased significantly during the time of culture (Figure 5B, black arrow), indicating the acquisition of fibro-chondrogenic differentiation in these cells. In addition, the differentiation potential of MSCs was confirmed by the evaluation of gene expression profile: a significant up-regulation of Aggrecan in the presence of M1 medium was observed in both ASCs and BMSCs, with levels increasing over time (Figure 6A). Similar trends were also observed in BMSCs for Collagen type I and type II expression (Figure 6B,C, right panel), whereas in ASCs we observed no induction for Collagen type I, and a different trend for Collagen type II expression (Figure 6B,C, left panel). Finally, in undifferentiated bioconstructs (SFM ASCs- and BMSCs-SCF), a significant effect of the collagen sponge used as a scaffold was observed: all the markers used to evaluate fibro-chondrogenic differentiation showed significant increases in SFM bio-constructs maintained for 7, 14, and 21 days compared to day 0 (Figure 7), suggesting the fibro-chondroinductive potential of collagen sponge on MSC populations.

### 2.4. Discussion

Surgical approaches for meniscal injuries are associated with a high risk of developing early osteoarthritis [27]. For this reason, over the last decades, tissue engineering approaches have gained popularity, thanks to the progress in cell biology, biomaterial development, and bioengineering. For the treatment of injured or damaged menisci, the use of fibro-chondrogenic cells in association with a suitable biomaterial is considered a promising alternative to the current standard of care. In this work, we have compared three cell sources in association with a collagen type I gel scaffold. The application of a collagen type I gel for meniscus tissue engineering has already been tested with promising results [28]. Moreover, a sponge scaffold was used for cartilage engineering in a rabbit model of chondral defect: authors found that after eight weeks post-implantation, newly formed cartilage appeared as typical mature cartilaginous tissue [29]. In our study, the microfibrillar structure increased the surface area for cell adhesion, thus providing a favorable environment for new cartilage formation in all the cell sources used. Mesenchymal stem cells are easily available with non-invasive procedures and can be extensively expanded in vitro to obtain a high number of cells to use in tissue engineering approaches. Because of their dual potential to act both as trophic mediators, capable of releasing anti-inflammatory and regenerative molecules, and to directly participate in tissue regeneration, MSCs are ideal candidates for tissue engineering approaches [30,31]. Bone marrow is the main cell source for adult MSCs along with adipose tissue which has been proposed as an alternative MSC source. Pre-clinical and clinical studies in which BMSCs were intra-articular injected [32,33] or used in association with scaffold [34,35] have been performed for the regeneration of the meniscus, suggesting a potential of these cells to delay the osteoarthritis progression and to support meniscus regeneration. On the other hand, a few studies demonstrated that ASCs could affect the healing rate of meniscal lesions in animal models [36,37]. At the same time, dermal fibroblasts represent another eligible cell source for meniscus engineering due to the simple procedure for their isolation and their high proliferation potential, which are important characteristics in tissue engineering approaches. Additionally, the literature has reported that they can upregulate collagen type II and proteoglycan when stimulated using scaffolds [38,39] or in 3D co-culture with chondrocytes [40]. Other studies, however, failed to demonstrate the chondrogenic differentiation of DFs even when cultured in a chondrogenic inductive medium [41,42]. In contrast, MSCs showed a good fibro-chondro-inductive potential when stimulated. The most promising growth factors from the TGF-β superfamily for cartilage tissue engineering, which we have also used in our experiments, are TGF-β1, TGF-β3, BMP-2, and BMP-7 [43]. TGF-β1 stimulates the synthetic activity of chondrocytes and acts with many regulatory activities on a large number of cells [44]. Experimental studies on MSCs have shown a down-regulation of collagen type I gene expression and an up-regulation of collagen type II and aggrecan gene expression [45,46]. TGF-β3 also induces cartilaginous ECM production, and the treatment of this cytokine on MSCs enhanced glycosaminoglycans synthesis [47]. Rui et al. also demonstrated that this process was significantly ameliorated by supplementation of BMP-2 (Bone Morphogenetic Proteins-2) [48], inducing an increase in cartilaginous ECM production correlated with a decrease in collagen type II expression. BMP-7 (also known as osteogenic protein-1, OP-1) plays an important role in cartilage regeneration, and on MSCs, it acts to decrease cell proliferation activity and stimulate the expression of cartilaginous ECM. An et al. demonstrated that the synergic effect of BMP-7 with TGF-β1 and IGF-1 (insulin-like growth factor-1) enhanced cell chondrogenesis [49]. In our study, we have demonstrated that both ASCs and BMSCs are sensitive to the treatment with members of the TGF-β superfamily. In particular, treatment with TGF-β3 and BMP-2 (M1 medium) induced the acquisition of fibro-chondrocyte-like cells, identifying them as adequate cell populations in meniscal tissue engineering approaches. Starting from these results, MSCs were used in association with collagen type I gel scaffold [50]. These gel scaffolds were also used in several studies and applications associated with different cell types, such as chondrocytes [51], bone marrow cells [52], oral keratinocytes [53], fibroblasts [54], and osteoblastic cells [55]. With the aim of identifying the best cell population to use in meniscus tissue engineering protocols, the behavior of ASCs and BMSCs and their differentiation potential toward a meniscal lineage in the presence of collagen type I gel were studied. Although either ASCs and BMSCs showed, in terms of viability and adhesion, good interaction with the scaffold, BMSCs presented a more linear differentiation trend, compared to cells derived from adipose tissue, with a constant and significant increase in all the analyzed marker characteristics of meniscal tissue (GAGs, aggrecan, collagen type I and type II). The positive effect obtained from the scaffold on undifferentiated MSCs (ASCs and BMSCs) is also notable: these cell populations demonstrate a great ability to respond both to chemical stimuli (cytokines and growth factors) and to physical stimuli supply of collagen sponge, confirming collagen as adequate support to promote cells viability and differentiation. Whitehouse et al. found similar results: the same collagen sponge seeded with autologous MSC was useful in a preliminary ovine study and later in a human study to repair torn avascular meniscus [56]. Other authors also observed an osteoinductive performance of the collagen sponge seeded using hMSCs derived from human adipose tissue [57].

In the future, an attractive objective could be to evaluate whether mechanical stimuli, provided by the use of an adequate bioreactor, will be able to standardize and enhance the differentiative performance toward a fibro-chondrogenic lineage of BMSCs and ASCs. Furthermore, calcium deposition and bone formation markers should be analyzed, considering the high bone differentiation potential of BMSCs. The easy availability, high cellular yield after isolation, and more pronounced proliferative capacity of ASCs compared to BMSCs, combined with their high propensity to respond to physical stimuli (scaffold and bioreactor), could make them the best cell source for meniscal tissue engineering approaches.

## 3. Conclusions

In conclusion, the present study demonstrated that mesenchymal stem cells (BMSCs and ASCs) and dermal fibroblasts (DFs) exhibit different potentials for meniscal tissue engineering. BMSCs showed the highest differentiation capacity, producing key meniscal markers when stimulated with TGF-β3 and BMP-2, while ASCs represent a valid alternative, mainly due to their easy accessibility and their less age-dependency [58,59]. Type I collagen scaffolds effectively supported cell adhesion and differentiation, particularly for BMSCs. The combination of mechanical and chemical stimuli through bioreactors could further enhance these regenerative approaches. In summary, choosing the right combination of cells and scaffolds is crucial for developing effective treatments for meniscal injuries and delaying the progression of osteoarthritis.

## 4. Materials and Methods

### 4.1. Cell Isolation and Characterization

Isolation and culture. Adipose-derived stromal cells (ASCs, *n* = 4), bone marrow stromal cells (BMSCs, *n* = 4), and dermal fibroblasts (DFs, *n* = 2) were isolated from the respective waste tissues deriving from healthy donors under informed consent and Institutional Review Board (IBR) authorization from Galeazzi Orthopaedic Institute, Milan, Italy. ASCs were isolated as previously described [60]. Briefly, tissues were enzymatically digested with 0.075% type I collagenase (225 U/mg; Worthington, Lakewood, NJ, USA) in a thermostatic chamber at 37 °C for 30 min. The stromal vascular fraction (SVF) was centrifuged, and 10^5^ cells/cm^2^ were plated in α-MEM medium (Table 2). BMSCs were isolated as reported by Torreggiani et al. [61]: cells were purified from aliquots of heparinized bone marrow aspirates and a Ficoll-Hypaque gradient (1.077 g/mL) (Sigma-Aldrich, Milan, Italy) was used. Nucleated cells were collected at the interface, washed twice, suspended in α-MEM medium, counted, and plated at a concentration of 10^4^ cells/cm^2^. For DFs, the tissue was mechanically minced, and the fragments were digested with 0.1% collagenase type I for 6 h at 37 °C. The digestion was filtered, centrifuged, and the pellet plated in CTRL medium (Table 2). All the cell populations were maintained at 37 °C in an incubator with 5% CO_2_. When cells reached 70–80% confluence, they were detached with 0.5% trypsin/0.2% EDTA (Sigma-Aldrich) and plated at a density of 5 × 10^3^ cells/cm^2^ for further expansions and experiments.

Proliferation. Cells were maintained in culture for several passages and counted every week. The proliferation rate was expressed either as a number of cells counted at each passage or as doubling time (DT) calculated as follows: t × ln(2)/ln(N/N0), where t is the time in culture (in hours), N is the number of harvested cells, and N0 is the number of seeded ones.

Clonogenic ability. Cells were plated in DMEM supplemented with 20% FBS, 100 U/mL penicillin, 100 μg/mL streptomycin, and 2 mM L-glutamine, in 6-well plates by serial dilution starting from 1000 cells/well. The frequency of the CFU-F was established after 10 days of culture (% CFU-F: number of colonies/number of plated cells × 100).

### 4.2. Cells Differentiation Assessment

Adipogenic differentiation. The density of 10^4^ MSCs/well and 5 × 10^3^ DFs/well was induced to differentiate into the adipogenic lineage using ADIPO medium (Table 2). After 14 days, samples were stained with Oil Red O solution (2% *w*/*v* Oil Red O in 60% isopropanol), and lipid vacuole production was quantified [60]. Absorbance was read at 490 nm.

Osteogenic differentiation. Cells were maintained either in control (CTRL) or osteogenic (OSTEO) medium (Table 2) at the density of 10^4^ MSCs/well and 5 × 10^3^ DFs/well. At 14 days, alkaline phosphatase (ALP) activity, collagen production, and calcium deposition were determined as previously reported [62]. Briefly, to evaluate alkaline phosphatase (ALP) enzymatic activity, both undifferentiated and differentiated cells were lysed in 0.1% Triton X-100 and incubated at 37 °C with 10 mM p-nitrophenylphosphate dissolved in 100 mM diethanolamine and 0.5 mM MgCl2, pH 10.5. Samples were read at 405 nm and ALP activity was calculated with respect to the protein concentration of each sample determined by BCA Protein Assay (Pierce Biotechnology, Rockford, IL, USA). To determine collagen production, cells were stained with 0.1% (*w*/*v*) Sirius Red F3BA in saturated picric acid (Sigma-Aldrich) for 1 h at room temperature, and then the stained samples were extracted with 0.1 M NaOH for 5 min. Absorbance was read at 550 nm, as previously described. A standard curve of known concentration of calf skin type I collagen (Sigma-Aldrich) was used to determine the concentration of secreted collagen. Extracellular matrix (ECM) calcification was determined on fixed cells stained by 40 mM Alizarin Red-S (AR-S, pH 4.1; Fluka). The mineral deposition was quantified by incubating the stained sample with 10% *w*/*v* cetylpyridinium chloride (CPC; Sigma-Aldrich) in 0.1 M phosphate buffer (pH 7.0) for 15 min to extract AR-S. Absorbance was read at 550 nm.

Fibro-chondrogenic differentiation. Two different fibro-chondro-inductive media (M1 and M2), with minor modifications from the literature [62,63], were used to perform the analysis (Table 2). The differentiation was obtained in pellet culture conditions: 5 × 10^5^ cells were centrifuged in a 1.5 mL centrifuge tube, the pellets were resuspended in serum-free medium (SFM, Table 2) or using M1 and M2 media, and then re-centrifuged; media were changed twice a week. After 21 days, pellets were analyzed by glycosaminoglycans (GAGs) production, histological analyses, and chondrogenic gene expression. Glycosaminoglycans (GAGs) production was assessed either by a qualitative analysis using Alcian Blue staining [64] or dimethyl methylene blue (DMMB) quantitative assay as previously described [65]. Briefly, for Alcian blue staining, micromasses were fixed with methanol 100% at −20 °C for 30 min and incubated with a 0.5% Alcian Blue Solution in 1M HCl overnight at room temperature. The staining was extracted using 6 M guanidine HCl in Milli-Q water for 6 h at room temperature. Absorbance was measured at 630 nm. For the quantitative assay, micromasses were digested at 56 °C overnight by 50 µg/mL proteinase K in 100 mM K_2_HPO_4_ (pH 8.0). After the inactivation of the enzyme for 10 min at 90 °C, the samples were spun at 14,000xg for 10 min, and each supernatant was collected for GAGs and DNA quantification. The samples were then incubated in 40 mM glycine/NaCl (pH = 3) with 16 mg/mL DMMB, and the absorbance was read at 500 nm. The amount of produced GAGs was determined with respect to a curve of known concentrations of chondroitin sulfate (Sigma-Aldrich) and normalized on total DNA content determined using the CyQUANT Cell Proliferation Assay Kit (Invitrogen, Milan, Italy), following the manufacturer’s instruction.

Histology. The samples were fixed in 4% buffered formalin for 24 h at room temperature, rinsed for 10 min in running tap water, and processed for paraffin embedding through a graded ethanol series; 4 µm-thick sections were obtained and then stained with Safranin-O following a standard protocol [66], for the evaluation of the structural details and GAG deposition.

Histometry. Scaffold and micromasses diameter length was estimated. The observations were made using an Olympus BX51 light microscope (Olympus; Milan, Italy), equipped with a digital camera.

Real-Time PCR. Total RNA was isolated from undifferentiated and differentiated cells using Trizol (Invitrogen) in accordance with the manufacturer’s instruction, and the isolated RNA was quantified spectrophotometrically (Nanodrop, Thermo Scientific, Rockford, IL, USA); 1 µg of RNA was reverse-transcripted to cDNA employing the iScriptcDNA Synthesis Kit (Bio-Rad Laboratories, Benicia, CA, USA), and 10 ng of cDNA was used as a template for real-time PCR (StepOne Plus system, Applied Biosystems, Foster City, CA, USA). TaqMan Universal PCR Master Mix and Assays-On-Demand kit for human Aggrecan (Hs00153936_m1), Collagen type I (Hs01076777_m1), and Collagen type II (Hs01060345_m1) were used. The mRNA levels of target genes were corrected for β-Actin mRNA levels (endogenous control). All PCR reactions were performed in duplicate for each sample.

### 4.3. Cells-Scaffold Interaction

Scaffold. ASCs and BMSCs were cultured with type I collagen Avitene™ Ultrafoam™ Collagen Sponge (Becton Dickinson Rowa, Milan, Italy;) and maintained until 3 weeks. Collagen-based Avitene Ultrafoam is primarily made from highly purified collagen derived from bovine sources. Moreover, the sponge-like structure provides a porous environment that can retain moisture and support cell infiltration.

Cell viability assay. 10^4^ cells were seeded in 96-well plates and cultured in the absence (MONO) or in the presence of the scaffold (SCAFFOLD, 0.2 cm Ø × 0.4 cm H) and monitored at days 1, 5, 9, and 14. An amount of 0.5 mg/mL of MTT (3-[4,5 dimethylthiazol-2-yl]-2,5-diphenyltetrazolium bromide, Sigma-Aldrich) was added, and cells were maintained for 4 additional hours at 37 °C. Formazan precipitates were solubilized by 100% DMSO (Sigma-Aldrich) and absorbance was read at 570 nm.

Cell adhesion. The adhesion of 2.5 × 105 cells on the scaffold (0.4 cm Ø × 0.4 cm H) was evaluated by DNA assessment at different time points (1–5–9 and 14 days). A pellet of 2.5 × 105 cells was used as a control. Scaffolds with cells were digested overnight at 56 °C by proteinase K (50 µg/mL in 100 mM K_2_HPO_4_, pH 8.0; Sigma-Aldrich), and DNA was measured by CyQUANT Cell Proliferation Assay Kit, as previously described.

Differentiation analysis. In total, 5 × 105 cells were seeded on collagen scaffolds (0.6 cm Ø × 0.4 cm H) and maintained for 7–14 and 21 days either in SFM or in M1 medium. At each time point the content of GAGs, histological analysis, and chondrogenic gene expression were evaluated as previously described.

### 4.4. Statistical Analysis

Data were analyzed using two-way analysis of variance (ANOVA) of the SAS (version 8.1, Cary Inc., North Carolina, USA), where culture medium and cellular type were the main factors (cell differentiation vs. adipogenic, chondrogenic and osteogenic lineages, and micro mass diameters). A two-way design was expanded to a three-way design with time as the third factor (scaffold chondrogenic differentiation and diameters). Values from each experimental sample were considered as the experimental unit of all response variables. The data are presented as least squared means ± SE. Differences between means were considered significant at *p* < 0.05.

## Figures and Tables

**Table 1:** Micromasses size used for the fibro-chondrogenic differentiation analysis. ***: *p* < 0.001.

Population	Micromasses Size (µm)
**ASCs SFM**	933.35 ± 28.83 × 878.18 ± 59.05
**ASCs M1**	963.25 ± 28.15 × 998.88 ± 50.13
**ASCs M2**	723.34 ± 27.46 × 780.43 ± 41.21
**BMSCs SFM**	625.49 ± 63.92 × 539.54 ± 162.93
**BMSCs M1**	1265.87 ± 90.45 × 1258.26 ± 120.52 ***
**BMSCs M2**	675.03 ± 37.39 × 621.66 ± 205.33
**DFs SFM**	678.87 ± 38.38 × 605.54 ± 15.83
**DFs M1**	571.20 ± 21.88 × 586.74 ± 40.09
**DFs M2**	670.98 ± 26.54 × 610.03 ± 26.62

**Table 2:** Specific media used either for cell expansion and maintenance (α-MEM, CTRL, and SFM) and for cell differentiation (ADIPO, OSTEO, M1, and M2). * Life Technologies (Milan, Italy); ^§^ R&D (Milan, Italy); ^£^ Sigma-Aldrich (Milan, Italy); ° CLS Behring (King of Prussia, PA, USA).

Medium	Composition	Growth Factors
Expansion Medium (α-MEM)	α-MEM * 10% FBS * 100 U/mL Penicillin * 100 µg/mL Streptomycin * 2 mM L-Glutamine * 100 mM HEPES buffer * 1 mM Sodium Pyruvate *	5 ng/mL FGF-2 ^§^
Control Medium (CTRL)	DMEM * 10% FBS 100 U/mL Penicillin 100 µg/mL Streptomycin 2 mM L-Glutamine 100 mM HEPES buffer 1 mM Sodium Pyruvate	
Adipogenic Medium (ADIPO)	CTRL medium	1 µMDexamethasone ^£^ 200 µM Indometacin ^£^ 500 µM IBMX ^£^ 10 µg/mL Insulin ^£^
Osteogenic Medium (OSTEO)	CTRL medium	0.01 µMDexamethasone 0.15 mM Ascorbic acid 2-phosphate ^£^ 10 nM Cholecalciferol ^£^ 10 mM β-glycero-phosphate ^£^
Serum Free Medium (SFM)	DMEM 100 U/mL Penicillin 100 µg/mL Streptomycin 2 mM L-Glutamine 100 mM HEPES buffer 1 mM Sodium Pyruvate 1× ITS (10 µg/mL insulin, 5.5 µg/mL transferrin, 5 ng/mL selenium) * 1.25 mg/mL Human Serum Albumine (HSA) °	
Fibro-Chondrogenic Medium (M1)	SFM medium	0.1 µM Dexamethasone 0.1 M mM Ascorbic acid 2-phosphate 10 ng/mL TGF-β3 ^§^ 10 ng/mL BMP-2 ^§^
Fibro-Chondrogenic Medium (M2)	SFM medium	0.1 µM Dexamethasone 0.1 M mM Ascorbic acid 2-phosphate 10 ng/mL TGF-β1 ^§^ 10 ng/mL BMP-7 ^§^ 200 ng/mL IGF-1 ^£^

## Data Availability

The raw data are available at this site: https://osf.io/dx5k8/?view_only=858624c5f79d4a78b9ec4de4a2b31a73.
